# Beware Weakening the Ivory Tower of MDT Diagnosis in Interstitial Lung Disease

**DOI:** 10.3390/jcm8111964

**Published:** 2019-11-14

**Authors:** Nazia Chaudhuri, Colm Leonard

**Affiliations:** 1 North West Lung Centre, Wythenshawe Hospital, Manchester M23 9LT, UK; nazia.chaudhuri@nhs.net; 2 Manchester Academic Health Science Centre, University of Manchester, Manchester M13 9PL, UK

Accurate diagnosis of interstitial lung disease (ILD) has always been the cornerstone of ensuring appropriate treatment planning and prognostic discussions with patients. However, the challenges of accurate diagnosis have always been significant, with increasing recognition of the key role of the specialist ILD multidisciplinary team (MDT) in achieving an accurate diagnosis [1,2,3,4]. In the United Kingdom, the emergence of licensed antifibrotic drugs for idiopathic pulmonary fibrosis (IPF) led to more referrals to ILD specialist centres and, hence, more patients receiving an MDT diagnosis and not just for IPF. However, with ILD, it sometimes seems that it can be one step forward and two steps back. With updated Fleischner and European Respiratory Society/American Thoracic Society guidelines on IPF diagnosis, it has felt like the clearer things become, the less—rather than more—certainty we have [1,2].

The availability of antifibrotic drugs pirfenidone and nintedanib has caused a change in MDT behaviour, with ILD that might previously have been labelled as non-IPF being designated as a ‘working diagnosis of IPF’. This may not be incorrect in the sense that a significant number of fibrotic CT scans atypical for IPF, would, on biopsy, reveal changes of IPF. A clinical phenotype with deterioration in face of steroid trial, even with a scan more consistent with nonspecific interstitial pneumonia (NSIP), may well be appropriate for antifibrotic drugs. However, there is a danger with this approach of ‘lumping’ all fibrotic disease into ‘suitable for antifibrotics’ and a resulting lack of focus on figuring out disease phenotypes and pathogenesis that could hinder further progress.

The European Respiratory Society (ERS) 2019 meeting in Madrid from September to October 2019 had a session on ‘lungs in the fog’, perhaps an appropriate title, with the speakers talking about the diagnostic difficulties separating out unclassified ILD, hypersensitivity pneumonitis (HP), and IPF. In some respects, we seem to have gone from obsessing over diagnostic accuracy and separating out ILDs to clinical trials that lump a whole range of ILDs, for example, the recent trial looking at antifibrotic treatment in progressive fibrosing ILD [5]. There is also a likely marketing authorisation for nintedanib in systemic sclerosis ILD. Are we entering an era where we switch off our diagnostic acumen as all fibrotic disease with potential to, or evidence of, progression will be directed to existing antifibrotic drugs?

The danger is that we enter a Chronic Obstructive Pulmonary Disease or Asthma scenario, where it has taken decades for respiratory specialist to understand and appropriately engage with separating out phenotypes within a disease label, rather than lumping them all together and allowing our brains to switch off.

An element of the risk of ‘lumping’ all fibrotic ILDs into ‘suitable for antifibrotic therapy’ is the potential devaluing of tissue biopsy. In the United Kingdom, a low percentage of patients have a video-assisted thoracoscopic surgical (VATS) biopsy, and caution around referral for VATS biopsy is warranted given the risks of death, exacerbation, and other morbidities associated with that procedure. However, the emergence of cryobiopsy as a useful diagnostic and research tool is at risk of being downgraded by the ‘lumping’ approach toward treatment decisions, which potentially devalues the role of tissue biopsy.

We therefore need to be careful not to fall into the downsides of the ‘lumping’ approach. It is laudable to have pragmatic approaches both clinically and in investigational trials to facilitate patients with a range of ILDs having access to new treatments that have disease-modifying potential, but we must redouble efforts to discover better biomarkers of the range of interstitial lung diseases (not just IPF) and, particularly, biomarkers (radiological, blood, lavage, genetic, even exhaled breath perhaps) that will aid in better understanding disease pathogenesis, open up potential new therapeutic pathways and, of course, to better inform discussions on prognosis with patients and optimise treatment decisions. Two papers in this issue emphasise the need for, and some of the work being done on new biomarker approaches [6,7]. The journey in ILD is only beginning!

## References

[1] Lynch D.A., Sverzellati N., Travis W.D., Brown K.K., Colby T.V., Galvin J.R., Goldin J.G., Hansell D.M., Inoue Y., Johkoh T. (2018). Diagnostic criteria for idiopathic pulmonary fibrosis: A Fleischner Society White Paper. Lancet Respir. Med..

[2] Raghu G., Remy-Jardin M., Myers J.L., Richeldi L., Ryerson C.J., Lederer D.J., Behr J., Cottin V., Danoff S.K., Morell F. (2018). Diagnosis of Idiopathic Pulmonary Fibrosis. An Official ATS/ERS/JRS/ALAT Clinical Practice Guideline. Am. J. Respir. Crit. Care Med..

[3] Barratt S.L., Creamer A., Hayton C., Chaudhuri N. (2018). Idiopathic Pulmonary Fibrosis (IPF): An Overview. J. Clin. Med..

[4] Kalchiem-Dekel O., Galvin J.R., Burke A.P., Atamas S.P., Todd N.W. (2018). Interstitial Lung Disease and Pulmonary Fibrosis: A Practical Approach for General Medicine Physicians with Focus on the Medical History. J. Clin. Med..

[5] Flaherty K., Wells A.U., Cottin V. (2019). Nintedanib in Progressive Fibrosing Interstitial Lung Diseases. N. Engl. J. Med..

[6] Skeoch S., Weatherley N., Swift A.J., Oldroyd A., Johns C., Hayton C., Giollo A., Wild J.M., Waterton J.C., Buch M. (2018). Drug-Induced Interstitial Lung Disease: A Systematic Review. J. Clin. Med..

[7] Sheu C.-C., Chang W.-A., Tsai M.-J., Liao S.-H., Chong I.-W., Kuo P.-L. (2019). Gene Expression Changes Associated with Nintedanib Treatment in Idiopathic Pulmonary Fibrosis Fibroblasts: A Next-Generation Sequencing and Bioinformatics Study. J. Clin. Med..

